# Percutaneous Transhepatic Stent Placement for Hepatic Venous Outflow Obstruction After Liver Transplantation: A Case Report

**DOI:** 10.7759/cureus.106081

**Published:** 2026-03-29

**Authors:** Sako Tomogane, Shun Goto, Toshiro Imamoto, Akira Maki, Masaya Miyazaki

**Affiliations:** 1 Department of Diagnostic Radiology and Nuclear Medicine, Saitama Medical Center, Saitama Medical University, Kawagoe, JPN; 2 Department of Emergency Medicine and Critical Care, Saitama Medical Center, Saitama Medical University, Kawagoe, JPN; 3 Department of Hepato-Biliary-Pancreatic Surgery and Pediatric Surgery, Saitama Medical Center, Saitama Medical University, Kawagoe, JPN

**Keywords:** case report, hepatic venous outflow obstruction, interventional radiology, percutaneous transhepatic approach, stent placement

## Abstract

Hepatic venous outflow obstruction (HVOO) is a serious complication after liver transplantation, and endovascular/vascular intervention is an effective treatment option. Most IR procedures for HVOO are performed via transjugular or transfemoral venous approaches, while the use of the percutaneous transhepatic approach has rarely been reported.

Here, we report a case of percutaneous transhepatic hepatic vein stent placement for HVOO after living donor liver transplantation (LDLT). A woman in her 50s who had undergone living donor liver transplantation two years ago was admitted to our hospital with weight gain and massive ascites. Contrast-enhanced computed tomography (CT) suggested HVOO, which was confirmed by CT arterial portography. Because selective catheterization of the hepatic vein via transjugular or transfemoral approach was unsuccessful, an ultrasound-guided percutaneous transhepatic approach using a two-step puncture technique with a 21-gauge fine needle was performed. Balloon venoplasty, followed by placement of two self-expanding stents, was performed across the stenotic segment. Hepatic venous outflow was restored immediately without procedure-related complications. Follow-up imaging demonstrated an improvement in hepatic congestion and sustained stent patency. This case highlighted percutaneous transhepatic hepatic vein stent placement as a useful alternative treatment for posttransplant HVOO when standard venous access is difficult.

## Introduction

Hepatic venous outflow obstruction (HVOO) is a serious complication after liver transplantation. The rate of HVOO occurrence after living donor liver transplantation (LDLT) is reported to be 2.3%-11.9%. The rate of HVOO occurrence becomes higher after LDLT than after deceased donor liver transplantation because of the complicated reconstruction of hepatic veins and small graft size. In cases of HVOO, hepatic congestion due to outflow tract obstruction results in symptoms similar to those of Budd-Chiari syndrome. Physical symptoms include abdominal pain due to hepatic swelling, massive ascites, splenomegaly, lower limb edema, and jaundice [1-5].

Diagnosis of HVOO is generally based on a combination of imaging findings and hemodynamic assessment. Contrast-enhanced computed tomography (CT) might show anastomotic stenosis of the hepatic vein or heterogeneous hepatic enhancement, which is suggestive of venous congestion. Hepatic venography with manometry can be used to confirm the diagnosis of HVOO by measuring the pressure gradient, which should be at least 5 mmHg across the stenotic site [6].

Interventional radiology (IR), including balloon venoplasty and stent placement, is currently the first choice for the treatment of HVOO [7, 8]. Most reported IR procedures for HVOO after LDLT are performed using a transjugular or transfemoral venous approach. However, in certain cases, selective catheterization of the affected hepatic vein through these conventional routes can be technically challenging because of severe stenosis or unfavorable vascular anatomy. In such situations, alternative access routes are required. Nevertheless, use of a percutaneous transhepatic approach for treatment of HVOO has not been well documented, particularly in adult LDLT recipients. Herein, we report a successful case of venoplasty and self-expanding metallic stent placement using an ultrasound-guided percutaneous transhepatic approach for HVOO after LDLT with some literature reviews.

## Case presentation

**Figure 1:**
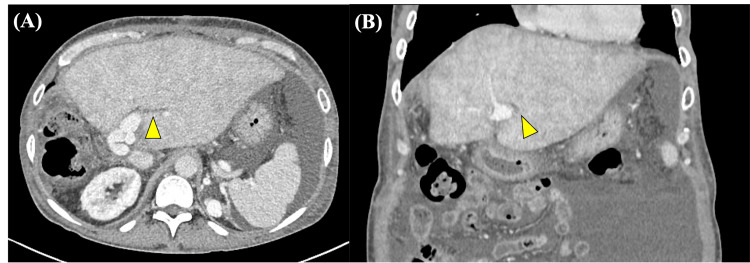
Contrast-enhanced CT images obtained in the portal venous phase Axial (A) and coronal (B) images demonstrate diffuse and heterogeneous hepatic enhancement with a periportal collar sign (arrowhead)

**Figure 2:**
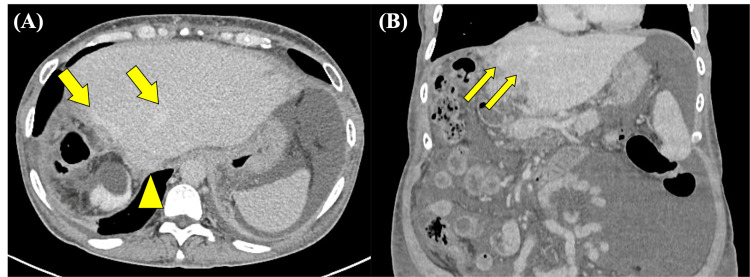
Contrast-enhanced CT images obtained in the venous phase Axial (A) and coronal (B) images show dilatation of the left and middle hepatic veins (arrows) relative to the inferior vena cava (arrowhead)

A woman in her 50s with primary biliary cholangitis had undergone living donor liver transplantation. During the transplantation procedure, after graft procurement and recipient hepatectomy, back-table venoplasty was performed. The left and middle hepatic veins were unified using a cryopreserved venous homograft in a collar configuration to create a large common orifice and prevent hepatic venous outflow obstruction, followed by vascular anastomoses and graft reperfusion. Two years after transplantation, the patient was admitted to our hospital with a chief complaint of weight gain of seven kilograms in one month and massive ascites. A liver biopsy revealed sinusoidal dilatation consistent with hepatic congestion. Portal-phase CT showed diffuse heterogeneous hepatic enhancement with a periportal collar sign (Figure 1). In the venous phase, contrast enhancement of the left and middle hepatic veins was greater than that of the inferior vena cava, suggesting HVOO (Figure 2).

**Figure 3:**
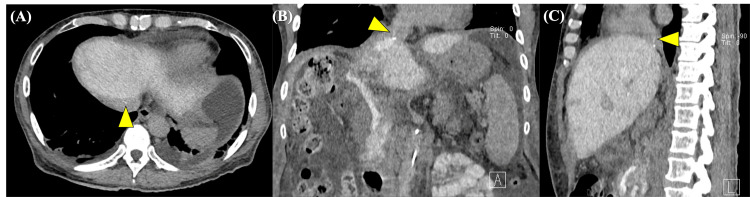
CT arterial portography images obtained during the second phase (80 seconds after contrast injection) Axial (A), coronal (B), and sagittal (C) images demonstrate sufficient contrast enhancement of the middle and left hepatic veins, with only a pinhole-like contrast passage (arrowhead) from the hepatic vein to the inferior vena cava

First, cavography was performed to confirm the diagnosis of HVOO. After skin preparation and local anesthesia, 5-Fr sheaths were placed in the right internal jugular vein and common femoral vein. Selective catheterization of the middle and left hepatic veins was attempted using 5-Fr cobra, shepherd-hook, and left adrenal vein catheters; however, catheterization via both venous approaches was unsuccessful. Subsequently, a 5-Fr sheath was placed in the right femoral artery, the superior mesenteric artery was selected, and CT arterial portography (CTAP) was performed in three phases (40, 80, and 120 seconds after contrast injection). In the second phase (80 seconds) of CTAP, sufficient contrast enhancement of the middle and left hepatic veins was observed; however, only a pinhole-like contrast effect from the hepatic vein to the inferior vena cava was observed (Figure 3).

Based on CTAP, the patient was diagnosed with HVOO. Percutaneous transhepatic venoplasty was planned because the transjugular or transfemoral venous approach was unsuccessful.

The patient remained clinically stable, and fluid management was performed with peritoneal drainage during the interval between cavography and treatment. The 13-day interval was attributable to the preparation of the equipment required for the percutaneous transhepatic venoplasty and scheduling constraints of the angiography suite.

**Figure 4:**
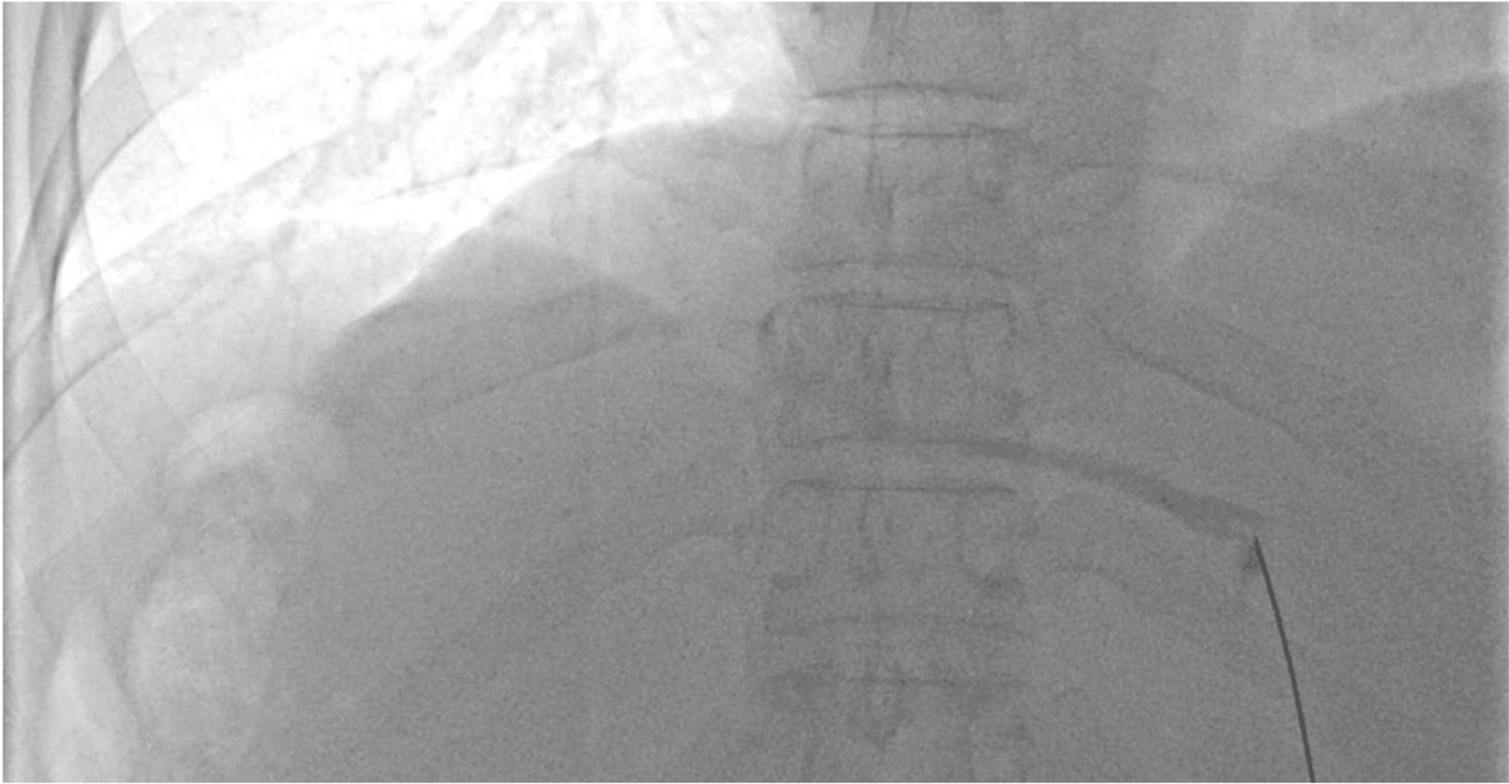
Angiography of the left hepatic vein. Ultrasound-guided percutaneous puncture of the left hepatic vein using a 21-gauge fine needle. Injection of contrast medium through the needle confirms opacification of the left hepatic vein.

Thirteen days after cavography, percutaneous transhepatic venoplasty was performed. We performed percutaneous liver puncture using a 21-gauge fine needle because puncturing a congested liver in patients with HVOO carries a high risk of bleeding. After skin preparation and local anesthesia, the left hepatic vein was punctured with a 21-gauge needle (PTCD needle; TOP Corp., Tokyo, Japan) under ultrasound guidance (Figure 4).

**Figure 5:**
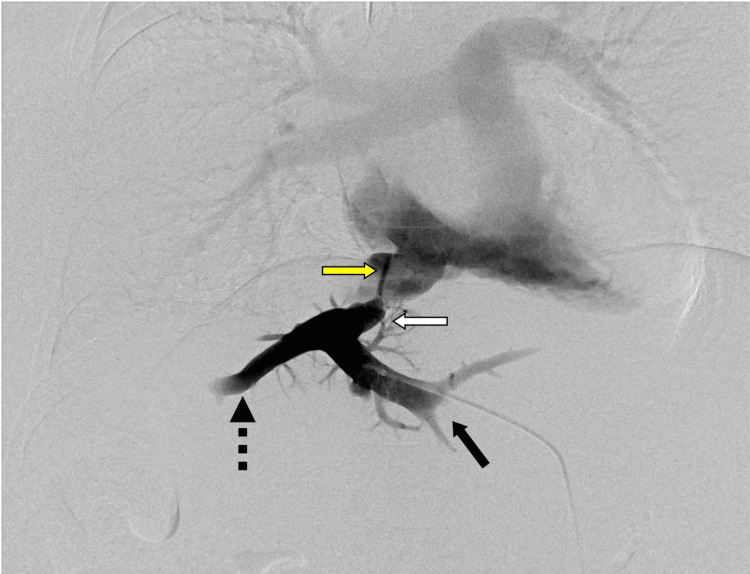
Digital subtraction angiography of the hepatic vein demonstrating a pinhole-like stenosis (yellow arrow) The anastomosis between the left (black arrow) and middle hepatic veins (black dotted arrow) and the homograft (white arrow) appears stenotic, with collapse of the homograft collar

After confirming correct needle placement by contrast injection, a 0.018-inch guidewire was introduced, and a 6-Fr triple-lumen introducer set (Neff access system; Cook Medical, Bloomington, IN, USA) was advanced over the guidewire. The guidewire was then replaced with a 0.035-inch angled hydrophilic guidewire (Radiforcus; Terumo, Tokyo, Japan), and the triple-lumen introducer was replaced with a 6-Fr sheath. Digital subtraction angiography (DSA) demonstrated severe pinhole-like stenosis in the proximal portion of the hepatic vein (Figure 5).

**Figure 6:**
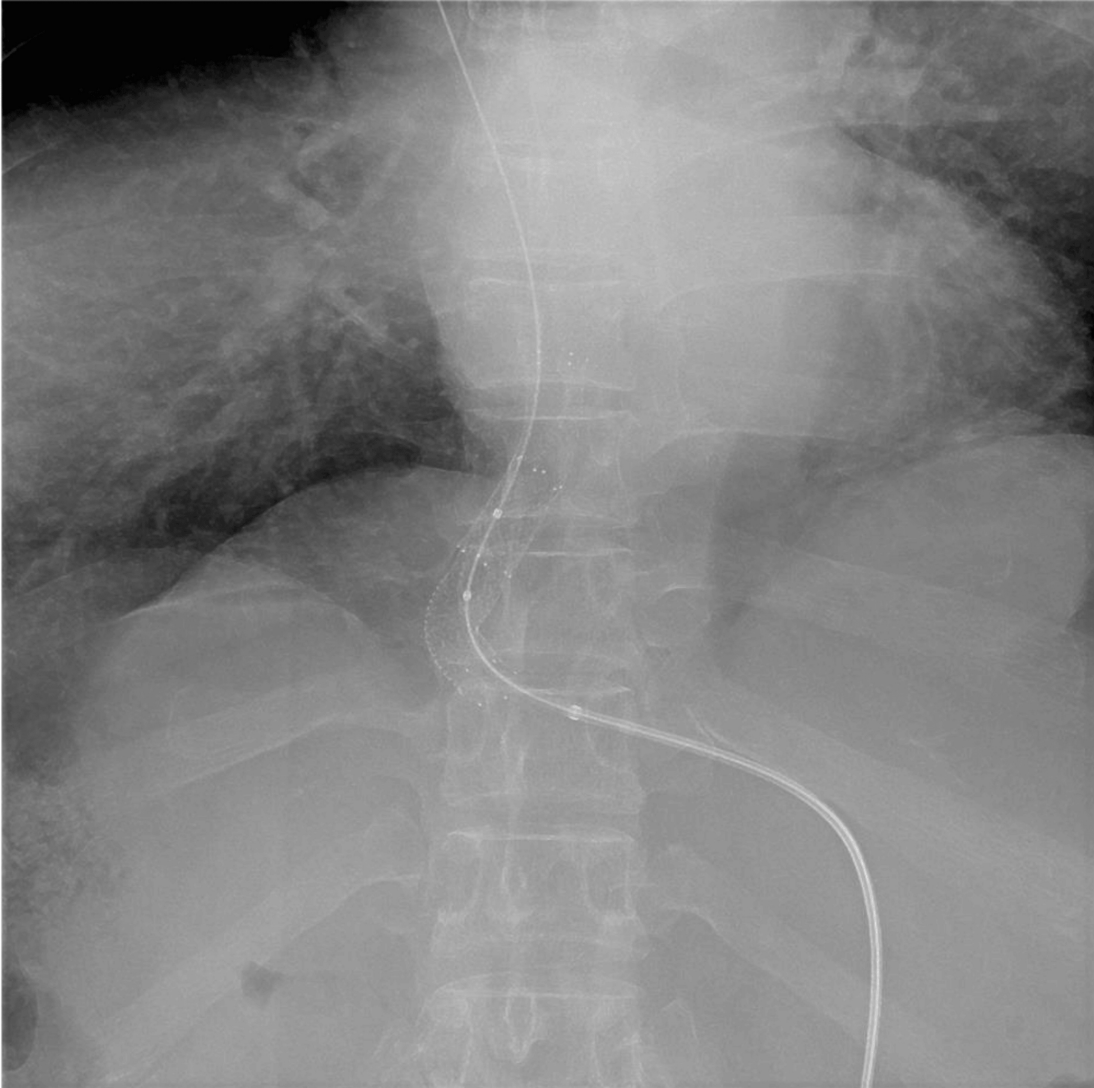
Two overlapping self-expanding stents (S.M.A.R.T. Control™ stent; Cordis, Miami Lakes, FL, USA), measuring 10 mm in diameter and 40 mm and 60 mm in length, are deployed across the stenotic segment

Passage through the stenotic segment was attempted using the guidewire (Radiforcus) and 5-Fr Cobra catheter (Medikit Co., Ltd., Tokyo, Japan). After passage of the catheter, venous pressure gradient across the stenosis was 14 mmHg before venoplasty. Balloon venoplasty was performed using a 6 × 40 mm angioplasty balloon catheter (Powerflex Plus; Cordis, Miami Lakes, FL, USA) with stepwise inflation at 6, 8, and 10 atm for 30 seconds each. Because only partial dilation was achieved, stent placement was subsequently performed. Two self-expanding stents (S.M.A.R.T. Control™ stent; Cordis, Miami Lakes, FL, USA) measuring 10 × 40 mm and 10 × 60 mm were deployed in an overlapping fashion across the stenotic segment (Figure 6). The initially deployed 10 × 40 mm stent migrated toward the right atrium relative to the stenotic segment. As a result, it did not sufficiently cover the target lesion, and an additional 10 × 60 mm stent was required to ensure complete coverage and adequate dilatation.

**Figure 7:**
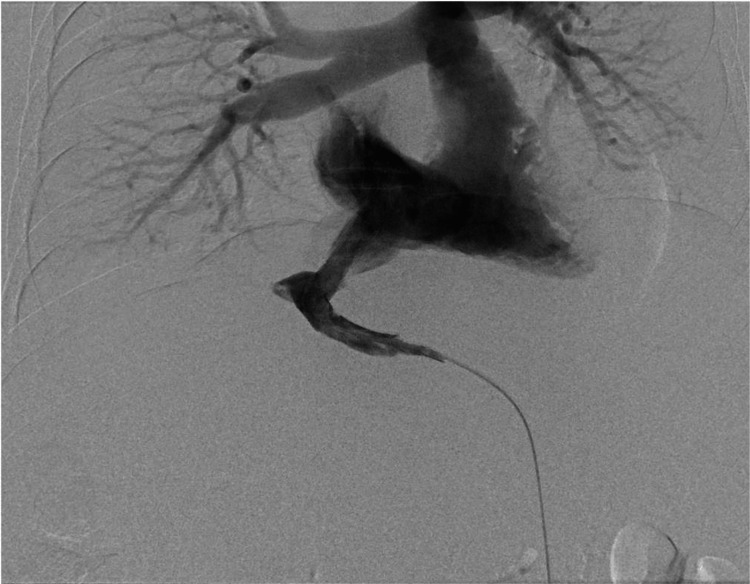
Post-dilation digital subtraction angiography Post-dilation digital subtraction angiography confirms full stent expansion and adequate dilation of the hepatic vein

After post-dilation using an angioplasty balloon catheter, DSA confirmed adequate stent expansion and restoration of hepatic venous outflow (Figure 7).

**Figure 8:**
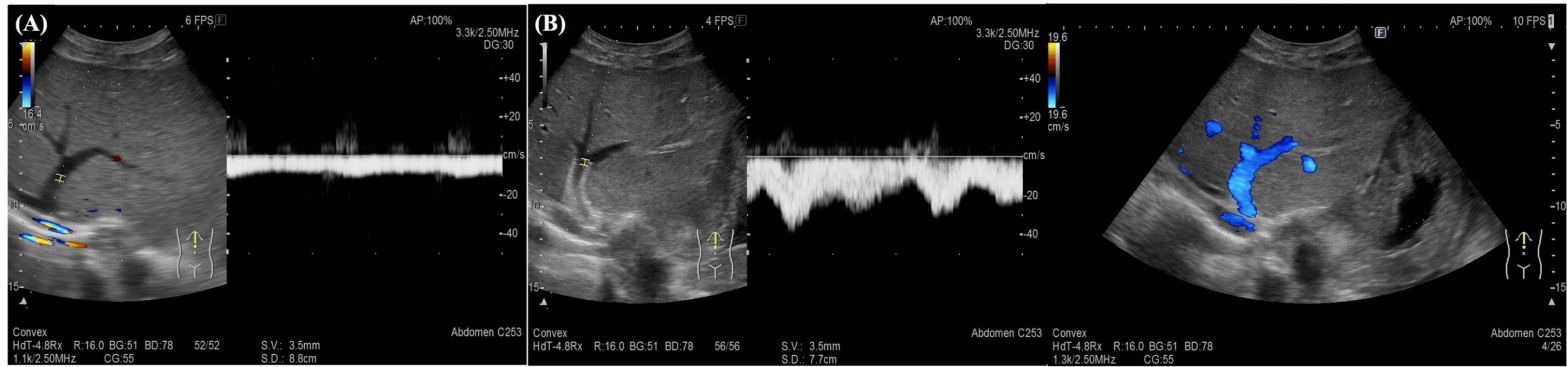
Doppler ultrasound findings of the left hepatic vein (A) Pre-treatment image demonstrating stagnated flow with loss of normal triphasic waveform; (B) Post-treatment image demonstrating restored venous flow with reappearance of the triphasic waveform

Venous pressure gradient significantly decreased to 2 mmHg after stent placement. Before the procedure, Doppler ultrasonography showed hepatic venous flow to be stagnated with loss of triphasic waveform (Figure 8A). After stent placement, triphasic waves and hepatic venous flow were restored (Figure 8B).

**Figure 9:**
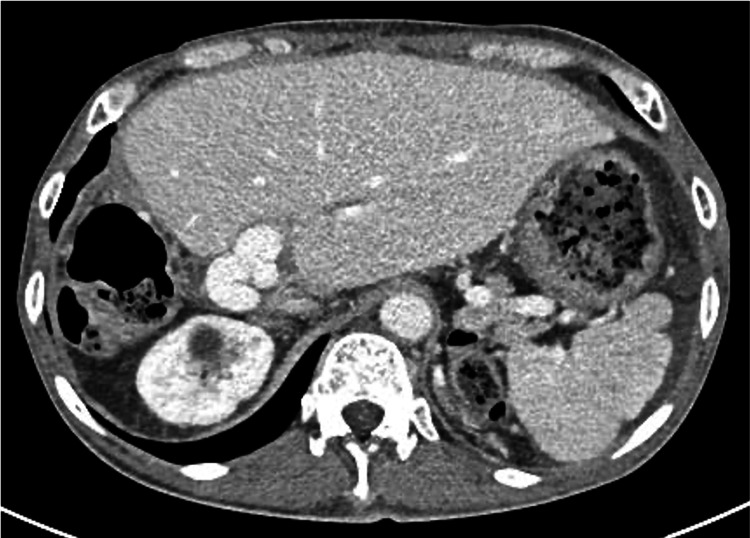
Contrast-enhanced CT obtained in the portal venous phase after stent placement Contrast-enhanced CT obtained in the portal venous phase after stent placement demonstrating resolution of diffuse and heterogeneous hepatic enhancement, indicating improvement of hepatic congestion

Follow-up CT demonstrated resolution of diffuse heterogeneous enhancement in the portal phase, indicating an improvement in hepatic congestion (Figure 9).

**Figure 10:**
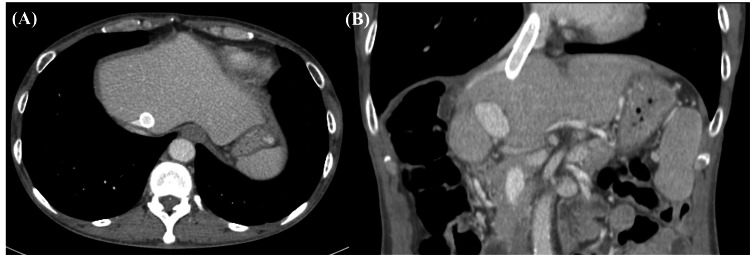
Follow-up contrast-enhanced CT images obtained 31 months after stent placement Axial (A) and coronal (B) images demonstrating patency of the stent in the left hepatic vein

The patient was discharged 16 days after stent placement without any procedure-related complications. Following the procedure, the patient's ascites resolved completely, and the seven-kilogram weight gain observed prior to treatment returned to baseline. No deterioration in liver function was observed before or after the procedure, and there was no elevation in bilirubin levels prior to treatment. A follow-up CT obtained 31 months after the procedure demonstrated sustained patency of the stent in the left hepatic vein (Figure 10).

## Discussion

IR has become a very effective treatment for HVOO after LDLT. Most patients who develop HVOO after LDLT undergo venoplasty or stent placement from either the jugular vein or common femoral vein approach [9]. However, venoplasty or stent placement using a percutaneous transhepatic approach has not been well documented.

Sambomatsu et al. reported findings on 15 adult patients treated via percutaneous transluminal approach through the right femoral vein or right internal jugular vein for HVOO after LDLT [9]. In general, when the target hepatic vein and site of anastomotic stenosis can be clearly identified, treatment via a transvenous approach can be performed safely and effectively. However, selective transvenous access may be technically challenging in cases of severe anastomotic stenosis or unfavorable vascular anatomy, as encountered in our patient. In such situations, an alternative access route via the percutaneous transhepatic approach should be considered. Although reports describing the percutaneous transhepatic approach for HVOO are scarce, Kubo et al. demonstrated that percutaneous balloon dilatation for HVOO after LDLT is safe and effective and suggested that the transhepatic approach may also be a viable option [10].

In the present case, we adopted a percutaneous transhepatic approach using a 21-gauge needle, which is conceptually similar to percutaneous transhepatic biliary drainage. Shimizu et al. reported that peripheral portal vein-oriented biliary drainage using a two-step guidewire method is safe and feasible in patients with non-dilated bile ducts [11]. Compared to standard cases, percutaneous transhepatic puncture in patients with HVOO carries a higher risk of bleeding due to hepatic congestion. Therefore, we believe that the percutaneous transhepatic approach using a 21-gauge fine needle may reduce the risk of complications such as hemorrhage. In our patient, no serious complications, including bleeding, were observed after direct liver puncture. While Kubo et al. reported using an 18-gauge needle for transhepatic puncture, the use of a 21-gauge needle, as in the present case, might contribute to safer treatment by minimizing puncture-related risks.

Whether balloon venoplasty or stent placement should be the primary treatment option for HVOO remains controversial. Several authors have recommended balloon venoplasty as the initial treatment [10, 12]. Kubo et al., argued against stent placement as the initial treatment for HVOO for the following three reasons, first, some patients can achieve sufficient patency with single-session balloon venoplasty, making stent placement unnecessary; second, many cases involve infants or children, in whom, long-term stent patency can be uncertain and vessel growth could lead to size mismatch; third, re-transplantation might be required in cases of disease recurrence or progressive rejection. In contrast, other studies have advocated stent placement, reporting a high rate of restenosis following balloon venoplasty alone [7, 13]. Ko et al. suggested that primary stent placement may be an effective solution for HVOO after LDLT in both adult and pediatric patients [13]. In addition, several reports have indicated that stent placement should be considered in patients who are refractory to repeated balloon venoplasty [9, 14, 15]. In the present case, stent placement was considered due to inadequate dilatation after balloon venoplasty.

Doppler ultrasound plays an important role in the diagnosis and follow-up of HVOO. Findings reported from Doppler ultrasonography include decreased hepatic venous flow velocity, distal hepatic vein dilation, and reduced portal venous flow [16]. In these, loss of the triphasic waveform of the hepatic vein is considered the most characteristic feature of HVOO. In our patient, preprocedural Doppler ultrasound demonstrated stagnation of hepatic venous flow with loss of the triphasic waveform. Following stent placement, Doppler ultrasound confirmed restoration of hepatic venous flow and recovery of the triphasic waveform, indicating hemodynamic improvement.

## Conclusions

HVOO after LDLT is an uncommon but potentially serious complication that can lead to graft dysfunction if not treated promptly. In this case, percutaneous transhepatic hepatic vein stent placement successfully relieved anastomotic stenosis when conventional transvenous access was technically difficult. The use of a 21-gauge fine needle allowed safe transhepatic access to congested livers without procedure-related complications. Doppler ultrasound and CT findings were useful for both diagnosis and post-treatment assessment. Percutaneous transhepatic stent placement should be considered as a safe and effective alternative approach for the treatment of post-transplant HVOO when standard venous routes are not feasible.
